# *β-*glucans: a potential source for maintaining gut microbiota and the immune system

**DOI:** 10.3389/fnut.2023.1143682

**Published:** 2023-05-05

**Authors:** Ravindra Pal Singh, Aditi Bhardwaj

**Affiliations:** Department of Industrial Biotechnology, Gujarat Biotechnology University, Gandhinagar, Gujarat, India

**Keywords:** *β*-glucans, gut microbiota, *Bifidobacterium*, *Bacteroides*, immune system

## Abstract

The human gastrointestinal (GI) tract holds a complex and dynamic population of microbial communities, which exerts a marked influence on the host physiology during homeostasis and disease conditions. Diet is considered one of the main factors in structuring the gut microbiota across a lifespan. Intestinal microbial communities play a vital role in sustaining immune and metabolic homeostasis as well as protecting against pathogens. The negatively altered gut bacterial composition has related to many inflammatory diseases and infections. *β-*glucans are a heterogeneous assemblage of glucose polymers with a typical structure comprising a leading chain of *β-*(1,4) and/or *β-*(1,3)-glucopyranosyl units with various branches and lengths as a side chain. *β-*glucans bind to specific receptors on immune cells and initiate immune responses. However, *β-*glucans from different sources differ in their structures, conformation, physical properties, and binding affinity to receptors. How these properties modulate biological functions in terms of molecular mechanisms is not known in many examples. This review provides a critical understanding of the structures of *β-*glucans and their functions for modulating the gut microbiota and immune system.

## 1. Introduction

Research of the current decade in the field of food science is paying close attention to studying microbiota and their associated health benefits (1). Diet is a critical modifiable factor and plays a crucial role in maintaining the microbiota and influencing their composition, proving the possibility of therapeutic dietary approaches to control microbial diversity, composition, and stability. The diet must also include non-digestible components, particularly dietary fibers, in addition to the necessary nutrients, including proteins, vitamins, lipids, and minerals, as diet strongly influences the composition of colonic microbiota and their metabolic products (2). *β-*glucans, a common component of the human diet, have several positive health effects (3, 4). Yeast, fungi (including mushrooms), certain bacteria, seaweeds, and cereals (oat and barley) contain *β-*glucans, and the polysaccharides of D-glucose monomers joined by *β-*glycosidic linkages (5, 6). *β*-glucans exist in different glycosidic linkages, such as β (1,3), (1,4), and (1,6) in either an unbranched or branched arrangement (7, 8). The abundant hydroxyl groups form hydrogen bonds with water which gives the molecule capacity to store water in both soluble and insoluble states, making it strongly hydrophilic. *β*-glucans’ molecular weight (MW) depends on the source and varies between 10^2^ and 10^6^ Da (9). High MW and high viscosity features of *β-*glucan cause hypocholesterolemia and hypoglycemia (10, 11). Notwithstanding, when *β-*glucans are used as a food factor, they are recognized for their capacity to change the functional aspects of food products, including viscosity, texture, rheology, and sensory qualities (12).

**Figure 1:**
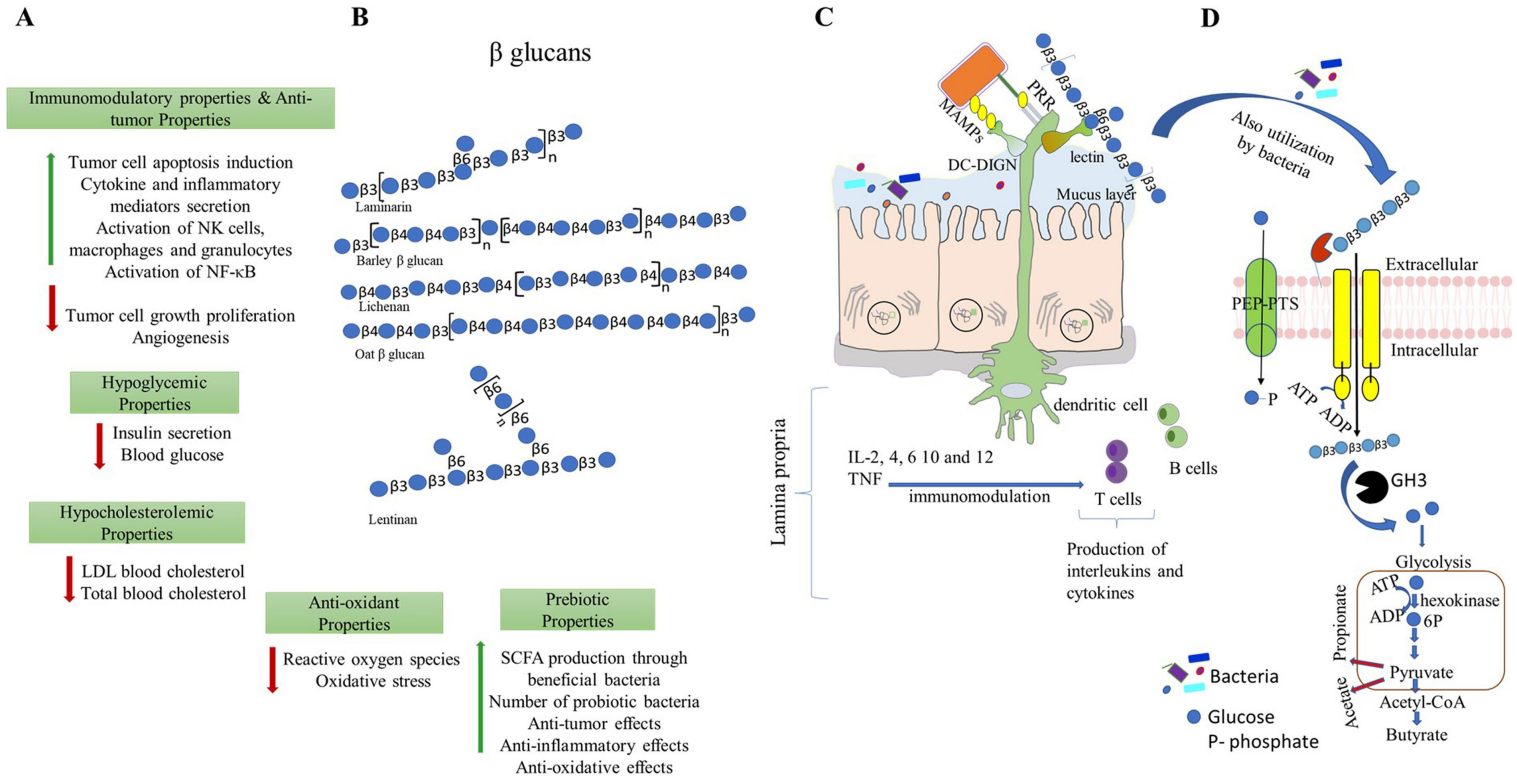
Different beneficial properties of *β-*glucans. **(A,B)** *β-*glucans can be extracted from different sources and have a wide range of applications for improving the health of the host. Representative structures of *β*-glucans are made as per symbolic nomenclature for glycans (https://www.ncbi.nlm.nih.gov/glycans/snfg.html). **(C)** *β-*glucans modulate immune responses by direct binding and recognizing receptors present in the immune cells. **(D)** *β-*glucans modulate gut microbiota, and in turn, they promote the production of short-chain fatty acids. PPR- pattern recognition receptors; MAMPs- microbe-associated molecular patterns and DG-SIGN- dendritic cell-specific intercellular adhesion molecule-3-grabbing non-integrin.

Because *β-*glucans are crucial to a healthy diet, the US Food and Drug Administration advises to consume 3 g *β-*glucan on a routine basis from cereal sources, such as barley or oats, to lower the risk of heart-related illnesses (13). Foods rich in *β-*glucans are a significant contender for a healthy diet due to their bioactive properties and numerous functional activities. Multiple features of *β-*glucan, including anticancer (14), anti-diabetic (15), anti-inflammatory, and a decrease in the glycemic index as well as serum cholesterol and triglycerides, have been demonstrated. *β-*glucans maintain the balance of blood glucose and cardiovascular diseases (16), enhance the immune system (17) and wound healing activities (18), and show antimicrobial (antibacterial and antiviral) properties (Figure 1). Proved antioxidant, wound healing, and moisturizing properties of *β-*glucan derived from microorganisms and cereal (19). These diverse activities of *β-*glucans attribute to their physical properties such as water solubility (20), viscosity, and gelation (21). Thus, the physical characteristics of bread and cakes enhance by adding *β-*glucan to the recipe (22).

*β-*glucans are known to show a robust immune stimulant (23) and mitigate benign and malignant cancers (24, 25). *β-*glucan acts as a pathogen-associated molecular pattern (PAMP) to inhibit the host’s insusceptible reactions (26). When a fungal infection occurs, the host recognizes a crucial PAMP associated with *β-*glucan and then stimulates the host’s immunological responses (27). Dendritic and macrophage cells are often regarded as the primary target cells of *β-*glucans that also stimulates neutrophils, B, T, and natural killer cells to attach pathogens (25, 28, 29).

Approximately 100 trillion microbial cells inhabit the gastrointestinal tract of the human, which encode 100 times more genes than the human genome (30). In addition, microbial cells are present in approximately 10 times the number of human cells (30, 31). This microbial community contains an estimated 5,000 bacterial species, mainly belonging to *Bacteroidetes*, *Actinobacteria*, *Firmicutes*, and *Proteobacteria* (31). The gut microbiota studies understand the status of different gut conditions between diseases and healthy for improving gut integrity, controlling host immunity, safeguarding the host against microbes, harvesting energy, and unraveling dietary utilizing molecular mechanisms (32, 33). The gut bacterial community plays a significant role in maintaining human wellbeing by producing many fundamental health-benefiting substances, including synthesizing vitamin K (34), promoting angiogenesis, involving host metabolic processes, and altering the appetite signaling pathway (35–38). Recent evidence also revealed that gut microbiota is critical in advancing cerebrum capabilities connecting with uneasiness, sorrow, stress, and cognizance (39, 40). There is always an association between gut dysbiosis and numerous chronic diseases (41), such as cardiovascular, autism, cancer, and obesity (42, 43). Dietary supplementation with indigestible polysaccharides obtained from plants, fungi, and probiotics benefits health by promoting microbial community growth (44). Bacterial fermentation occurs when dietary fibers enter the colon (45), and as a result, they produce short-chain fatty acids (SCFAs such as acetic acid, propionic acid, and butyric acid) (46), biogenic amines (47), indole and tryptophan derivatives (48), and secondary bile acids, conferring a health benefit. Microbial-produced SCFAs play significant parts in the proliferation of immune cells, apoptosis, cell differentiation, chemotaxis, and gene expression (49). Bile acids (50) and tryptophan derivatives (48) also play a gene regulatory role. The gut microbial communities harbor several unique genes that encode different enzymes to break down carbohydrates, including glycoside hydrolases, amino acid decarboxylases, carbohydrate esterases, and polysaccharide lyases (51). These highly diverse thousands of carbohydrate-active enzymes (CAZymes) of microbial communities are referred to as the host’s secondary genome (52). Therefore, the usage of dietary fibers entirely depends on CAZymes.

Gram-negative microorganisms, sjuch as *Bacteroides*, possess many glycoside hydrolases and polysaccharide lyases (35), while Gram-positive bacteria, such as *Lactobacillus* and *Bifidobacterium* (36), primarily have glycoside hydrolases for breaking down different types of dietary fiber (53). How these microbes utilize dietary fiber and their produced metabolites modulate the immune system are outstanding questions among glycobiologists and biochemists. However, there are few known molecular mechanisms for digesting *β*-glucan by bacteria and structure-functional interactions between β-glucan and the immune system. The latest understanding of them is summarized here.

## 2. Microbial composition in the human GI tract and capability for digesting the dietary fiber

Microorganisms in the intestine of humans fluctuate from a few hundred to 100 trillion, such as 10^3^ cells/g in the stomach, ~ 10^7^ cells/g in the small intestine with a more significant part of facultative anaerobes, and ~ 10^13^ cells/g in the colon which obligate anaerobes make up the majority fraction (54, 55). The gut microbiome populace overwhelmingly incorporates the individual from phyla *Bacteroidetes* (primarily *Bacteroides* and *Prevotella*), *Firmicutes* (primarily *Clostridia genus*), *Fusobacteria*, *Actinobacteria*, and *Proteobacteria* (56, 57). *Bacteroidetes* and *Firmicutes* are the major phyla that account for approximately 90% of the total bacteria in the adult gut. *Bacteroidetes* and *Proteobacteria* regulate the immune system, formation of the gut microbiome, and defense against pathogen invasion (58). They maintain the microbiome and immune systems through an integrated metabolic energy-harvesting process based on dietary fiber cross-feeding (syntrophy) and co-metabolism, including polyphenols (36, 59).

Individuals from the *Bacteroidetes*, a predominant phylum in the human gut, have polysaccharide utilization loci (PUL) to focus on a wide variety of complex glycans. The arrangement of genes centered on tandem susC/susD homologs that code for the TonB-dependent transporter (TBDT) and the cell-surface glycan-binding protein (SGBP) (60). Extra colocalized and co-regulated SGBP(s), susC/susD, and a transcriptional regulator typically make up machinery that detects, imports, and upregulates a PUL in the presence of glycans (61). Only a few PULs have been now biochemically characterized in *Bacteroidetes*, despite a massive number of them being recognized (62). For utilizing *β-*(1,3)-glucans, bacteria use activities of *β-*(1,3) glucanases (EC 3.2.1.6 and EC 3.2.1.39) and *β-*(1,3) glucosidase (EC 3.2.1.58) that have a place with the glycoside hydrolase families, GH5, GH16, GH17, GH55, GH64, GH81, GH128, and GH158 (63). *β-*(1,3)-glucanases break-down the glycan with internal glucoside bonds and make oligosaccharides. *β-*(1,3)-glucosidases act on the non-reducing ends of *β-*(1,3)-glucans and discharge glucose from oligosaccharides (64). Some endo-acting *β-*(1,3)-glucanases have carbohydrate binding domains to enhance their capacity to bind substrates that are not soluble in water (65), while *β-*(1,6)-glucanase (EC 3.2.1.75), a member of the GH30 family, is necessary to dissect *β-*(1,6) linked branched chains (33).

## 3. Impact of *β-*glucan on gut microbiota

Diet is a major factor in regulating the diversity and activity of gut microbiota, including how ingested diet is shared among the microbial communities at different syntrophic levels. This interaction determines the balancing of the gut microbiota and preventing of non-communicable diseases (66, 67). Indeed, *β-*glucan is a non-digestible carbohydrate and acts as a substrate for improving colonic microbes as they are permitted to go through the small intestine due to their resistance to absorption (68, 69). Several studies on *β-*glucans that modulate gut microbiota are presented in Table 1.

**Table 1:** Modulation of gut microbiota by *β-*glucans.

Source of *β-*glucan *Study model*	Molecular weight or/and composition of material that was used	Modulation of Gut microbiota	References
*Cereal-β-glucan*			
Tibetan hulless barley-*in vitro* fermentation of human fecal	3.45 × 10^4^ Da	↑ *Pantoea, Megamonas, Bifidiobacteria, Prevotella*↑ Concentrations of SCFA (acetate, propionate, butyrate)	Nie et al. (70)
Barley *in vivo* - human patient with high risk for metabolic syndrome development	Experimental *β-* glucans bread was prepared with wheat flour and *β-* glucans-enriched barley flour (Valechol)	↑ *Bifidobacterium* spp. and *Akkermansia municiphila*	Velikonja et al. (71)
Barley-*in vivo* in rat	LMW- *β-* glucans were partially prepared by cellulase MW 12 kDa	↑ *Bifidobacterium* and *Bacteroides* ↑ Total SCFAs, particularly ↑ Acetate and n-butyrate.	Aoe et al. (72)
Barley-*in vivo hypercholesterolemic rat*	LMW barley	↑ *Bifidobacterium*	Mikkelsen et al. (73)
Barley-*in vivo* study on human	Granoro’s Cuore Mio pasta was made by using a mixture of durum wheat flour (75%) and whole-grain barley flour (25%)	↑ *Roseburia hominis, Ruminococcus* ssp. *Clostridiaceae* spp.↓ *Fusobacteria* and *Firmicutes*	De Angelis et al. (74)
Barley-*in vivo* randomized individual study	HMW barley (1,349 kDa)	↑ *Bacteroidetes* and ↓ *Firmicutes*	Wang et al. (75)
Oat-*in vitro* fermentation of colonic microbiota	PepsiCo, Inc. (Barrington). Used different oat ingredients	↑ *Bifidobacterium, Roseburia, Lactobacillus* spp.	Van den Abbeele et al. (76)
*Mushroom-* *β-* *glucan*			
*Pleurotus eryngii in vitro* – fermentation of human fecal sample	Mushroom cultivation was conducted in substrates consisting of wheat straw or beech sawdust, and in their mixtures in various ratios (w/w) with grape marc or olive prunings and of olive leaves with two-phase olive-mill waste. Therefore, used mushroom may have contamination of used substrates.	↑ *Bifidobacterium* spp. and *F. prausnitzii* populations.↑ Acetate, propionate and butyrate concentration.	Boulaka et al. (77)
*Fungal* *β-glucan*			
*Grifolan (Grifola frondosa)-in vitro-* colorectal cell lines		↑ *Lactobacillus* and *Bifidobacterium.* ↑ Lactic, succinic, and valeric acid concentrations.	De Giani et al. (78)
Polysaccharide form *Ganoderma lucidum-in vivo* obesity mice model	MW: 133.1 KDa	↑ Ratio *Bacteroides* to *Firmicutes*, *Bacteroides ovatus* and *B. uniformis*. ↑ Acetic, propionic, butyric and valeric acid.	Chang et al. (79)
*Yeast* *β-glucan*			
Zymosan-*in vitro* fermentation model		↑ *Bifidobacterium, Faecalibacterium, Prevotella*↓ *Escherichia-Shigella*↑ Acetic acid and propionic acid	Pi et al. (80)

Cereal and oat-β-glucan: Mixed-linkage (1,3) and (1, 4)-linked β-D-glucans, and fungal-β-glucan: (1,3) and (1, 6)-linked β-D-glucans.

*β-*glucan increases the growth of *Lactobacillus casei, Lactobacillus acidophilus,* and *Bifidobacterium animalis* subsp. *lactis* both *in vivo* and *in vitro* (81). Cereal *β-*glucans were fed to experimental rats for 3, 6, and 7 weeks, and the results showed that the population of *Bifidobacterium* and *Lactobacillus* was enhanced (82). *Clostridiaceae* (*Clostridium orbiscindens* and *Clostridium* sp.), *Roseburia hominis, Ruminococcus* sp., and low levels of *Firmicutes* and *Fusobacteria* were found to be more abundant after the consumption of whole grain barley pasta and durum wheat flour rich in *β-*glucan (74). In yogurt, *β-*glucans of oats and grains were found to expand the development and reasonability of *Bifidobacterium animalis* subsp*. lactis* (83). An increased gut-bacterial population has positive functional consequences, such as ensuring adequate digestion and preventing constipation, diarrhea, and inflammatory bowel disease (IBD) (84, 85). In addition, integrating high molecular weight oat *β-*glucan into milk brings cholesterol down and decreases calories in dairy items (86). Oat and barley-β-glucans increase beneficial bacterial communities’ population and promote microbial metabolites such as 2-methyl-propanoic, butyric acid, propionic acid, and acetic acid (87–89).

Bacterial communities of our gut reveal the diverse digestion capability of dietary glycans. For instance, *Bifidobacterium* cannot digest complex glycan, such as pectin. Thus, they rely on *Bacteroides* to produce oligosaccharides from pectin before they can grow. This type of cooperation is known as a syntrophic system, and different bacteria have adjusted their genome through evolution to maintain gut microbial homeostasis (90). *Bifidobacterium* and *Bacteroides* are essential members of the gut microbiota, where they occupy approximately 80% of microbial space in infant and adult gut, respectively, involving in the utilization of dietary glycans. Therefore, *Bacteroides* and *Bifidobacterium* are primary and secondary degraders for utilizing complex and simpler glycans, respectively. Some of the known glycan-utilizing mechanisms are mentioned below pertaining to *β-*glucans.

### 3.1. Trapping of *β-*glucan by some gram-positive and gram-negative human gut bacteria

Gram-negative bacteria, such as *Bacteroidetes,* can access and grow on a broad spectrum of complex glycans, which they encounter in the gastrointestinal tract of humans (91). As aforementioned, they comprise a starch utilization system (Sus), which is a hallmark distributed across their phylum (92). PULs have enabled human gut *Bacteroidetes* to utilize xylan (93), arabinoxylan (94), rhamnogalacturonan I (95) and II (96), and various other plant polysaccharides (97, 98), and their detailed molecular mechanism has been characterized by comprehensive functional analyses.

The degradation of *β-*glucan mainly occurs extracellularly by outer membrane-bound enzymes which produce oligosaccharides upon digestion of complex polysaccharides. Utilization of oligosaccharides by Gram-negative bacteria depends on an outer membrane protein complex consisting of an extracellular SGBP and an integral membrane SusC-like TonB-dependent transporter. Crystal structures of two practically distinct SusCD complexes purified from *B. thetaiotaomicron* have derived a standard model for substrate translocation (99). The TBDT forms homodimers, with each *β-*barrel protomer tightly capped by SGBP. The single-channel electrophysiology revealed a ‘pedal bin’ mechanism in which SGBP (SusD homolog) moves away from TBDT (SusC homolog). In the absence of oligosaccharides, the SusD lid of the empty transporter is free and undergoes conformational changes. In the presence of glucan, TonB binds to the TonB box of the transporter to initiate the conformational changes in the plug, extracellular loops of SusC, that lead to oligosaccharide release and the creation of a transport channel into the periplasmic space (99). The TonB promotes the dissociation of glucan into periplasmic space, and then, the transporter (SusC) returns to its open state conformational. The required energy is governed by ExbBD–TonB system, which is equivalent to pressing the pedal to open the SusCD (99). These mechanistic insights into how the outer membrane nutrients are imported inside the periplasm and cytoplasm by microbiota members provide outlines of understanding human–microbiota symbiosis.

**Figure 2:**
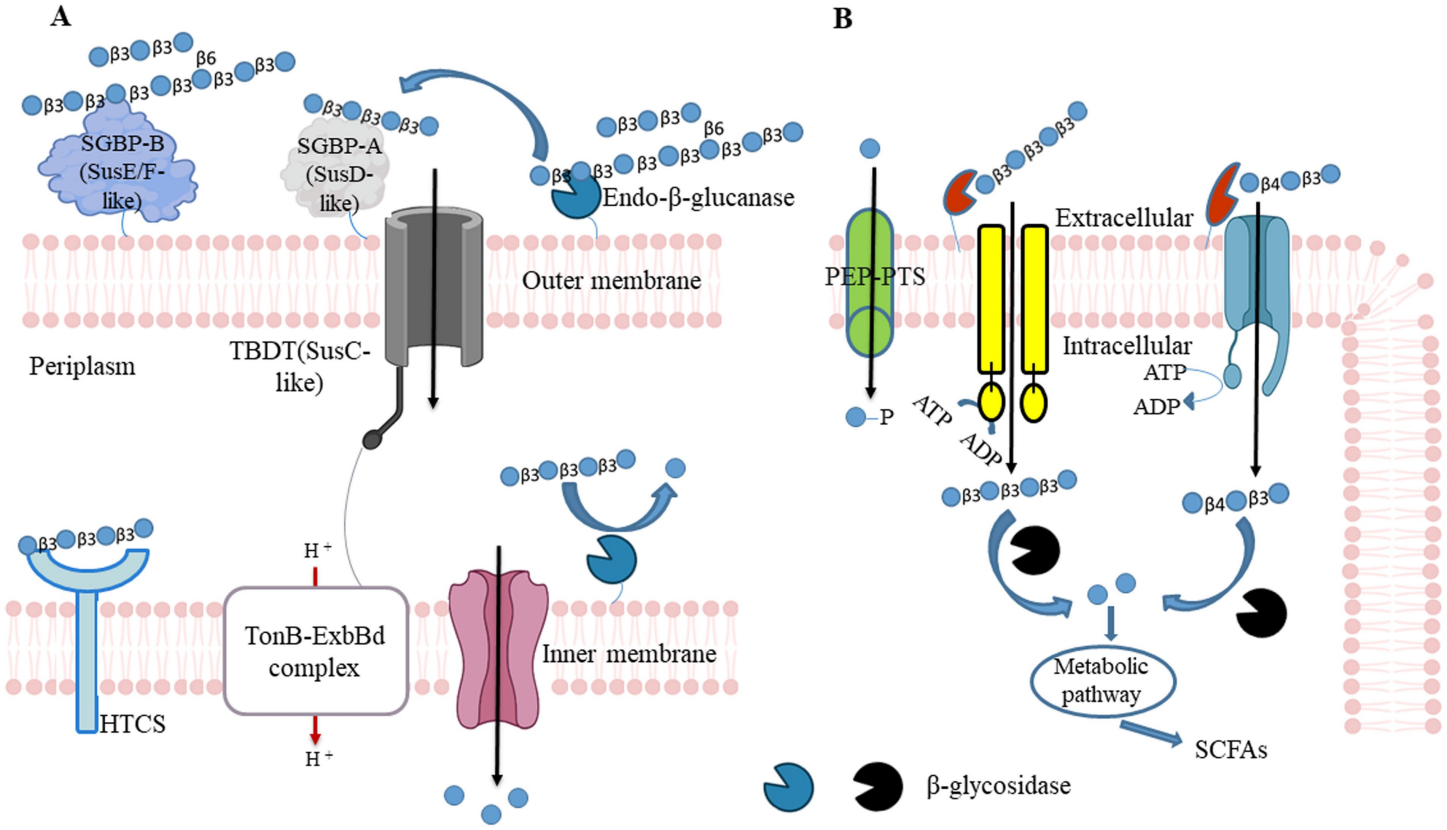
*β-*glucan utilizing mechanisms in Gram-negative bacteria and Gram-positive. **(A)** Gram-negative bacteria can hydrolyze polysaccharides at the outer membrane tendered glycoside hydrolase (GH) and convert into oligosaccharides. Those polysaccharides first recognize by surface glycan-binding protein and facilitate endo-acting enzymes to cleave them. Generated oligosaccharides are again caught by SusD, and it allows them to enter the periplasmic space through TonB - dependent transporter (SusC homolog). Entered oligosaccharides further cleave into monosaccharide contents by periplasmic GH. Those monosaccharides pass to the cytoplasm *via* Major Facilitator Superfamily (MFS) transporter and produce bacterial metabolites (including SCFAs) through fermentation to promote the host’s health. HTCS, hybrid two-component system sensor/regulator. **(B)** Similarly to Gram-negative bacteria, Gram-positive bacteria hydrolyze polysaccharides at the outer membrane tendered GH and convert into oligosaccharides. Generated oligosaccharides enter into cytoplasmic space through MFS, the phosphoenolpyruvate (PEP)- carbohydrate phosphotransferase system (PTS), or ATP-binding cassette (ABC) transporters couple ATP hydrolysis. SCFAs: short-chain fatty acids.

For example, a mechanism for the utilization of barley-β-glucan was established in *B. ovatus* ATCC 8483 and *B. uniformis* JCM 13288 (61, 100). These studies demonstrated through synteny analyses that the mixed-linkage glucan utilization locus (MLGUL) is widely present among human gut microbiota. The presence of homolog genes within or without locus enables selective *Bacteroides* species in the gut microbiota to cleave barley-*β-*glucan. The locus consists of outer membrane-bound GH16 and a periplasmic GH3 that acts as exo-*β-*glucosidase. The GH16 cleaves high MW barley-*β-*glucan and produces mixed-linkage *β-*(1,3)/*β*-(1,4) glucan-oligosaccharides. Those oligosaccharides are converted into monomeric units by GH3 in periplasmic space. The GH3 is a part of the locus or is present in another site of a genome in *B. ovatus* ATCC 8483 and *B. uniformis* JCM 13288, respectively (61, 100). Locus outer membrane-bound non-catalytic SGBPs plays essential roles in recruiting and capturing high MW barley-*β-*glucan, and the SusD allows mixed-linkage *β-*(1,3)/β-(1,4) glucan-oligosaccharides to enter in periplasmic space in concert with cognate TonB-dependent transporters (TBDTs) as shown in Figure 2.

Polysaccharide degradation is accomplished by Gram-positive bacteria by different cellular mechanisms that may not involve TonB-dependent transporter systems. These symbionts encode polysaccharide degrading functions with genetic loci that encode ATP binding cassette (ABC), proton symporters, or phosphoenolpyruvate phosphotransferase system (PEP-PTS) transporters. These are co-expressed with associated degradative enzymes (101, 102). Oligosaccharides are internalized by ABC transporter-coupled ATP hydrolysis serving as the primary transport system in *Bifidiobacterium*. In PEP-PTS, phosphoenolpyruvate serves as the phosphate donor to the recipient monosaccharide, and PTS internalizes monosaccharide and concomitant phosphorylation. Although these systems are present in various bacteria, all *Bifidiobacterium* do not possess them in their genomes.

Interestingly, *g*ram-*p*ositive *p*olysaccharide *u*tilization *l*oci (gpPULs) are present in families of *Roseburia* and *Eubacterium rectale* that ideally consist of ABC transport proteins, transcriptional regulator genes, and glycoside hydrolases (103). Such gpPULs are found to involve in the utilization of konjac glucomannan and spruce acetylated galactoglucomannan (104) and xylan and arabinogalactan utilization (103). We have identified *β-*glucan utilizing gpPUL in *Blautia producta* ATCC 27340 and widely presence in many species of *Lachnospiraceae* (98). Distinct from Gram-negative bacterial PUL, gpPUL does not encode SGBP, but the glycan-binding function is likely to perform by carbohydrate-binding modules associated with endo-acting enzymes. The gpPUL also encodes a transcriptional regulator gene that seems to control the expression of the locus in the presence of suitable carbon (105).

In a symbiosis system between *Bacteroides* and *Bifidobacterium,* it was observed that *Bacteroides cellulosilyticus* and *Bacteroides ovatus* share *β-*(1,3)/(1,6)-glucooligosaccharides with *Bifidobacterium breve* UCC2003 and *Bifidobacterium bifidum* (102). Zhao and Cheung (106) suggested that *B. infantis*, *B. longum*, and *B. adolescentis* can ferment *β-*glucans obtained from mushroom sclerotia, seaweed, bacteria, and barley. Among them, *B. infantis* produces double amount of SCFAs than other two *Bifidobacterium.* However, a systematic evaluation of *β-*glucans utilization is required to use species for mitigating gut-related syndromes through appropriate modulation.

### 3.2. *β-*Glucan sensing by bacteria

The capability of gut *Bacteroidetes* to reckon and respond to diverse glycans in their environment is bestowed in many extracellular sensor-regulator systems that are closely associated with the PUL they encode. The most biochemically and structurally well-characterized system in *Bacteroidetes* is the hybrid two-component system (HTCS) (107). HTCS is a cytoplasmic membrane-spanning protein that comprises all domains of a classical two-component system in one polypeptide (N-terminal extracellular sensor, cytoplasmic histidine kinase, and response regulator). Signal recognition in HTCS takes place *via* the direct binding of oligosaccharide fragments to the periplasmic sensor domain. These oligosaccharides are products of polysaccharide degradation at the outer membrane cell surface-tethered PUL-encoded endo-acting enzymes. The produced oligosaccharides were earlier transported into the periplasm by the TBDT (SusC homolog). In some cases, oligosaccharides process further *via* periplasmic enzymes before acting as activating signals (108).

Although the sensing system in Gram-positive bacteria is not extensively known as in gram-negative bacteria, there are few transporters known to mediate glucan uptake and can readily utilize them through highly conserved sequences of the solute binding protein (Figure 2B). For instance, in *Bifidobacterium animalis* subsp. *lactis* to overcome the need for HTCS has the presence of an ABC transport system that allows the tethering and uptake of complex glycan such as arabinoxylan. The solute-binding protein, such as *Bl*AXBP, of ATP-binding cassette (ABC) transporter mediates the uptake of arabinoxylan–oligosaccharides with exceptionally broad specificity for tri-saccharides and tetra-saccharides of undecorated xylo- and arabinose-decorated-oligosaccharide (109). Crystal structures of *Bl*AXBP suggested that a spacious binding pocket and the conformational flexibility of a lid-like loop facilitate the binding of decorated oligosaccharides. The *Bl*AXBP is highly conserved within *Bifidobacterium* and highlights the gut microbiota metabolic syntrophy with other species. The occurrence of transport systems is a prerequisite for utilizing glucan- oligosaccharides and xylooligosaccharides. Solute-binding protein is also identified in *Limosilactobacillus reuteri* ATCC 53608 and *Blautia producta* ATCC 27340 for utilizing xylooligosaccharides (105).

The expression of the locus or gene involved in utilizing available carbon sources is suppressed by the presence of a preferred glycan. It is controlled by carbon catabolite repressor (CCR), a regulatory system in most bacteria (92). It is accomplished by different regulatory mechanisms, including the regulator of translation by an RNA-binding protein in diverse bacteria. The CCR-related metabolism was first seen in *B. animalis* subsp. *lactis* (110). It was also observed in other members of *Bifidobacterium* that can control the expression of genes involved in the utilization of raffinose, sucrose, or oligofructose (111).

The mechanism by which a glycan is utilized by *Bifidobacterium* is not yet well established as it is known for *Bacteroides*. Due to their usage in probiotics, detailed emphasis should be given to how specific genes/enzymes sense, break-down, and import complex glycans inside the cytoplasmic space by gram-positive bacteria. Novel pathways from *Bifidobacterium* would clearly elucidate the metabolites that play a role in maintaining gut homeostasis. The members of *Lachnospiraceae* express a gpPUL that consists of the transcriptional regulator (103, 104); however, the defined function of the such regulator is not yet known. The function of such a regulator should exploit by further studies.

## 4. Immunomodulatory effects of *β-*glucan

The gut microbiota constantly interacts with the immune system aiding diverse processes such as behavior, digestion, as well as the maturation of the immune system (Table 2); thus, it shows a symbiotic relationship with the host (138, 139). The immune system is also acknowledged as one of the most critical factors that affect the composition of the gut microbiota through cross-talk between immunity and microbiome (140). The colonization of gut microbiota can mediate and influence the production of antimicrobial peptides/bacteriocins through epithelial cells and pattern recognition receptors encoded by intestinal layers (141).

**Table 2:** Immunological studies of-*β-*glucan.

Type of *β-*Glucan	Source	Immunomodulation effects/properties *in vitro* and *in vivo*	References
Lantinan	*Lentinula edodes*	Enhances the phenotypic and functional maturation of dendritic cells with significant IL −12 productions.	Wang et al. (112)
		Reduction in anti-inflammatory cytokines such as IL-4, IL-10. It significant increases weight gains, blood cells, monocytes, circulatory cytotoxic T-cells. It increases in cage-side health of acute myeloid leukemia demonstrated in animal studies in Male BN/RijHsd rats.	McCormack et al. (113)
		Increases NK cell-mediated killing of Yac-1 cells both *in vitro* and *in vivo*.	Vetvicka et al. (114)
		Enhances cytotoxic activity and inflammatory cytokines of macrophages and RAW 264.7 cell lines.	Chan et al. (115)
		Increases anti-tumor activity in BALB/c mice inoculated with S-180 cells.	Zhang et al. (116)
		Lentinan-activated macrophages and dendritic cells indirectly activate T cells *via* IL-12 and IFN-γ.	Murata et al. (117)
		Increases T cell functions in cancer patients.	Yoshino et al. (118)
Laminarin	Fronds of *Laminaria*	The increased population of B, T and macrophage cells due to the administration of laminarin in the normal mice as compared to BALB mice, demonstrated in *in vivo* studies.	Shang et al. (119)
	*Laminaria digitata*	Induces anti-cancerous effect by activating dendritic cells, antigen-specific T cells in the C57BL/6 rodents, and releases pro-inflammatory cytokines such as TNF-α, IL-12 and IL-6 in B16 melanoma cells.	Song et al. (120)
	*Laminaria digitata*	Enhancement in the expression of IL-6 and IL-8 in response to *ex vivo* LPS-induced in pigs due to 600 ppm dietary inclusion of laminarin.	Smith et al. (121)
	Brown algae	Interleukin (IL-6 and IL-1β) and TNF-α have been expressed in RAW 264.7 cells under *in vitro* conditions.	Lee et al. (122)
		Induces apoptosis *via* Fas pathway and blocks the insulin-like growth factor-I (IGF-1, which has a role in cancer development) receptor in human colon adenocarcinoma H29 cells.	Park et al. (123)
		Strong binding efficiency for Dectin-1 in macrophages isolated from C57BL/6 mice under *in vitro* conditions.	Brown et al. (124)
Zymosan	*Saccharomyces. cerevisiae*	Activates TLR 2 and Dectin-1 on macrophages.	Dennehy et al. (125)
		Increases cytokine production such as TNF-α and IL-12 *via* NF-kB signaling. Increases production of monocyte chemo-attractant protein-1.	Lebron et al. (126)
Schizophyllan	*Schizophyllum commune*	Increases the expression of cytokines and activity of NK cells.	Yoneda et al. (127)
	Fungal Schizophyllan	Inhibited spread of the virus in the lungs. Augmented protective immune responses induced by low doses of a live *Sendai virus* vaccine. It was determined through animal studies.	Hotta et al. (128)
Polysaccharide ganoderma	*Ganoderma lucidum*	Increases MAPKs and Syk-dependent TNF-α and IL-6 expressed in CHO cells RAW264.7 cells. It also increases anti-tumor activity.	Guo et al. (129)
		It induces human peripheral blood mononuclear cell proliferation and produces cytokines like IL-10 and IL-12.	Chan et al. (115)
Pleuran	*Pleurotus ostreatus*	Increases proliferation of lymphocytes.	Mitsou et al. (130)
PGG glucan	*Saccharomyces cerevisiae*	Induces activation of NF-κB like nuclear transcription factor in purified human neutrophils, and enhances neutrophil anti-microbial function.	Wakshull et al. (131)
Algal *β-*glucan	*Durvillaea antarctica*	Increases activation of CD19+ B lymphocytes under *in vitro* studies.	Bobadilla et al. (132)
Phycarine	Seaweed	Stimulate both humoral and cellular branches of immune reactions to cure gastrointestinal diseases under *in vitro* studies.	Vetvicka et al. (133)
	*Laminaria digitata*	Significantly stimulates phagocytic activity in animal studies.	Vetvicka and Yvin (134)
Scleroglucan	*Sclerotium rolfsii*	Increases in TNF-α in human monocytes.	Falch et al. (135)
Ulvan	*Ulva intestinalis*	Releases cytokines such as IL-1β, IL-4, IL-6, IL-10, IL-11, IL-12, IL-13 and TNF-α, and activation of RAW 264.7 cells under *in vitro* conditions.	Tabarsa et al. (136)
		Expresses anti-tumor activity as inhibited the cell growth of breast cancer cell line by the *U. lactuta*. It decreases the anti-apoptotic marker (BCL-2) and tumor suppressor gene (P53) under *in vitro* conditions.	Lahaye and Robic (137)

In addition to the interaction of immunity and microbiome, *β-*glucans are considered one of our diet’s active ingredients that show immunological benefits. They can interact with various immunological receptors, including Dectin-1, complement receptor (CR3), and toll-like receptors (TLR) 2/6. This causes several immune cells to be triggered, such as dendritic cells, macrophages, neutrophils, monocytes, and natural killer cells (142). *β-*glucans can modulate innate and adaptive responses, and they can also improve opsonic as well as non-opsonic phagocytosis (143, 144). The intricacy of their structure governs diverse *β*-glucan immune functions. Stronger immune-modulating and anti-cancer actions are correlated with higher structural complexity (145). The direct binding of *β-*glucans to particular immune cell receptors raises the possibility of an immunological modulatory action independent of microbes (146).

**Figure 3:**
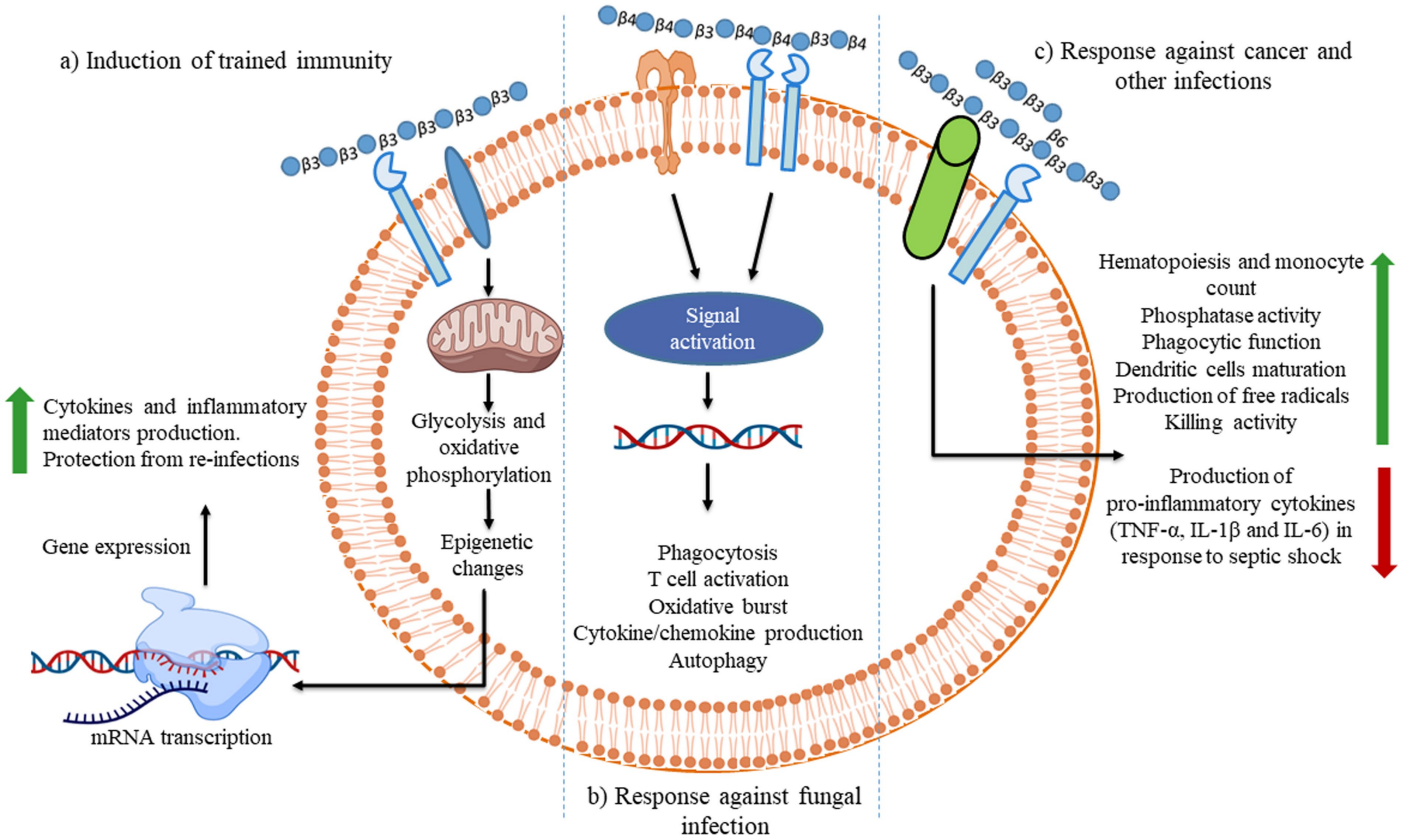
Illustration of the different consequences of *β-*glucan recognition by immune cells in the context of fungal infection. The recognition of different *β-*glucans triggers innate immune memory.

The ability of an innate immune system to rapidly recognizing and reacting to invasive pathogens is crucial for infection control. *β-*glucan guards against illness brought on by bacteria, viruses, and other harmful microbes (147). During *in vivo* investigations, *β-*glucans were tagged with fluorescein to monitor their oral uptake and digestion (148). The orally administered *β-*glucans bind to the Dectin-1, a type II transmembrane *β-*glucan receptor, on the macrophages and get taken up by the cell (149). It was demonstrated that low MW *β-*glucans bind strongly with Dectin-1 as compared with HW ones (150). It was subsequently moved to the bone marrow, lymph nodes, and spleen. Large *β-*(1,3)-glucans get degraded by macrophages within the bone marrow and produce smaller, soluble *β-*(1,3)-glucan fragments (Figure 3). This soluble *β-*(1,3)-glucan fragments were then recognized through CR3 of the circulating monocytes, macrophages, and granulocytes (151). These granulocytes with CR3-bound *β-*glucan-fluorescein when enrolled to a site of complement activation were enabled CR3 to trigger cytotoxicity of inactivated complement 3b (iC3b)-opsonized tumor cells, covered in monoclonal antibodies (mAb) (148). Yeast *β-*(1,3)/(1,6)-glucan and barley *β-*(1,3)/(1,4)-glucan potentiated the action of anti-tumor mAb, leading to more robust tumor regression and survival (148). When a lentinan, a type of *β-*glucan, binds to dectin-1, it activates Syk kinase that regulates COX2 expression, modulating immune responses.

Lentinan isolated from the fruiting bodies of *Lentinus edodes* is a popular medication with anti-infective and anti-tumor activities. RAW264.7 cell line’s cytotoxic activities and inflammatory cytokine production were enhanced by lentinan (29, 152). The dendritic cells show enhanced phenotypic and functional maturation and produce a considerable amount of IL-10 and IL-12 due to the presence of lentinan (115). Lentinan acts as a vaccine adjuvant, enhancing the virus-specific CD8(+) T cell functions generated by DNA vaccination in HBcAg (pB144) in mice (112) and raising T cell functions in mice with tumors (113) and malaria-infected mice. Lentinan-induced dendritic and macrophage cells indirectly activate T cells by producing IL-12 and IFN-γ (117). Lentinan increases NK cell-mediated death of Yac-1 cells in both *in vitro* and *in vivo* experiments (114).

**Figure 4:**
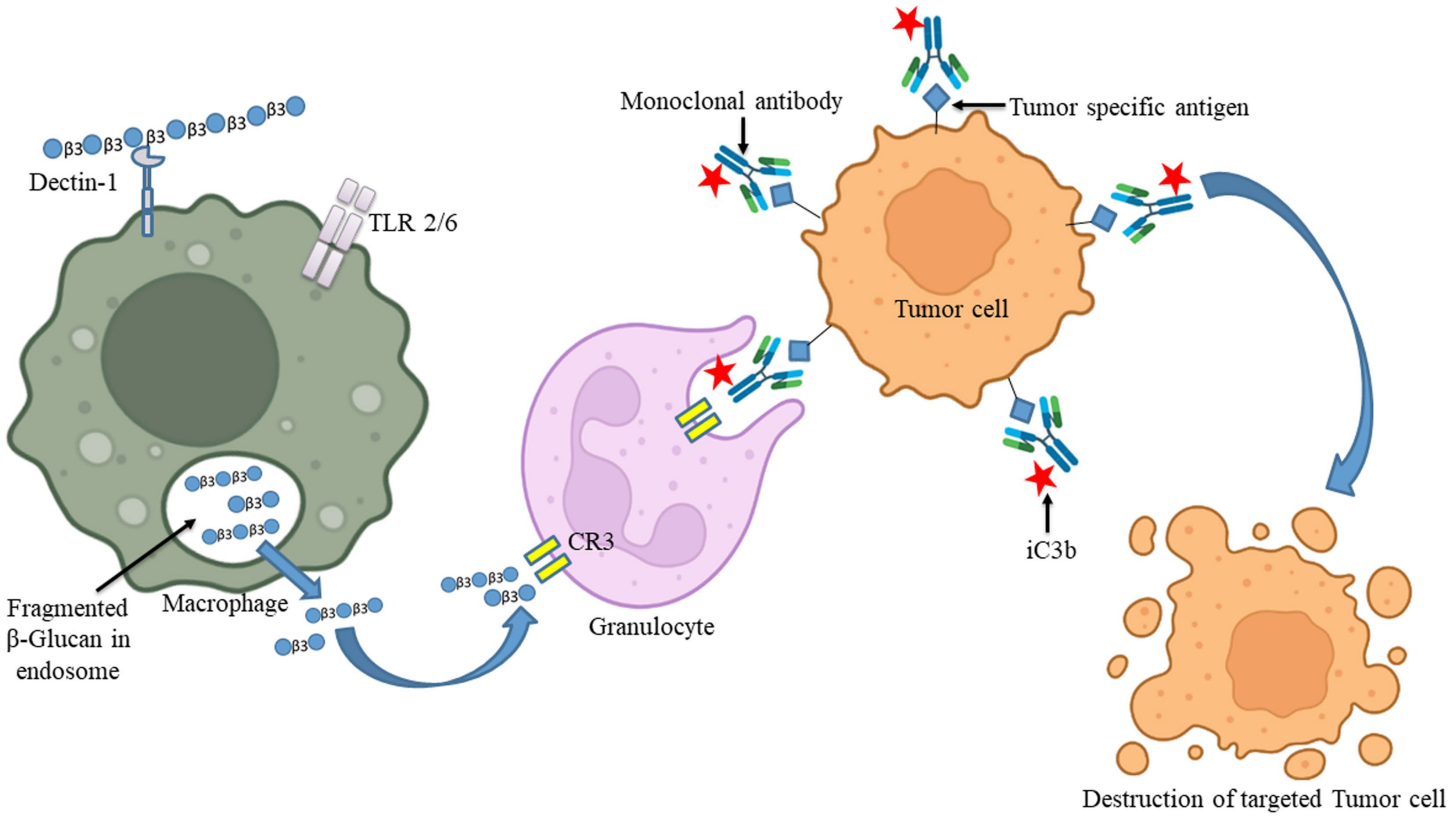
The uptake of *β-*glucan polysaccharides by macrophages and subsequent actions of *β-*glucan- oligosaccharides on immune cells. *β-*glucans are captured by macrophages through Dectin-1/TLR-2/6. The polysaccharides form of *β-*glucans gets internalized by the macrophages. Afterward, those are fragmented into oligosaccharides, which are subsequently released from macrophages. The circulating granulocytes eventually take these oligosaccharides by the complement receptor (CR)-3. The immune response will then be turned on and will be released by several monoclonal antibodies. Those released monoclonal antibodies have destroyed monoclonal antibody-tagged tumor cells. Images were prepared in BioRender.

**Figure 5:**
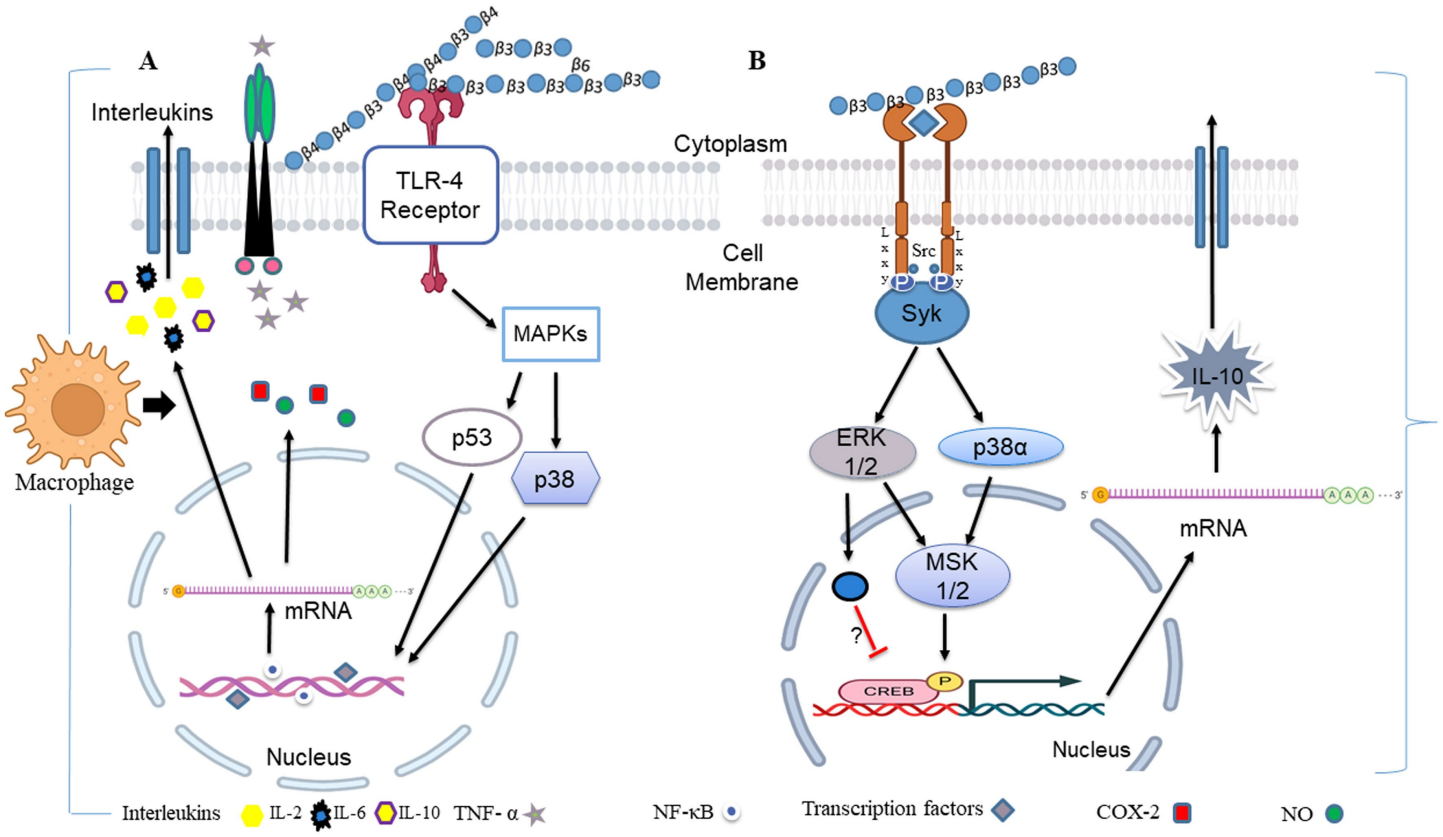
During fungal pathogen infection, the innate immune system recognizes fungal *β-*glucans as pathogen-associated molecular patterns through Dectin-1 and toll-like receptor 4. **(A)** Dectin-1 activation by zymosan was shown to promote the production of pro-inflammatory cytokines IL-6, IL-8 and TNF-α and anti-inflammatory IL-10 in macrophages *via* co-binding with TLR 2/4 through NF-κB signaling. **(B)** Dectin-1 receptor forms clustering when it binds with *β-*glucans and subsequently forms a phagocytic synapse. It allows Syk to bind at ITAM like the cytoplasmic domain of dectin-1. It then activates downstream signaling, including p38α MAPK and the ERK1/2 cascades. Both p38α and ERK1/2 phosphorylate and activate the protein kinases (MSK1 and 2). MSK1 and 2 switches on CREB through phosphorylation on the IL-10 gene promoter that promotes IL-10 mRNA transcription. In addition, ERK1/2 also inhibits IL-10 mRNA transcription *via* MSK and p38 independent pathways, although this mechanism is not precisely known yet (154). Dectin-1 also activates NF-κB, which inducts IL-2, 6, and 10 transcriptions. Produced cytokines activate monocytes and circulatory cytotoxic T-cells (113). Activates circulatory cytotoxic T-cells to destroy cancerous and fungal pathogenic cells through MAPKs signaling and p53 (155).

In contrast to the Dectin-1-Cox2 signaling axis, mannan/*β-*(1,6)-glucan-containing polysaccharides (MGCP) facilitate regulatory T (Treg) cell differentiation from naïve T cells. Additionally, it confines Th1 differentiation of effector T cells based on a TLR2-dependent mechanism through suppressing IFN-γ expression. Thus, the administration of MGCP exhibits a strong suppressive capability toward investigational colitis and autoimmune encephalomyelitis in mice models. It highlights the potential therapeutic utility of MGCP against clinically related autoimmune diseases (153). *β-*glucan-based immunological responses that trigger through receptors are summarized below as demonstrated in Figures 3–5.

### 4.1. *β-*Glucan receptors

Pattern recognition receptors (PRRs) are the typical cell surface receptor possessed by immune cells, including macrophages and dendritic cells that recognize PAMPs and other naturally occurring ligands, such as *β-*glucans (156). Dectin-1 and toll-like receptor (TLR) are major PRRs for *β-*glucans (157). Several receptors, such as CR3, scavenger receptors (SR), Dectin-1, the TLR, and lactosylceramide (LacCer) are involved in recognizing *β-*glucans. When these receptors connect to *β-*glucans, a signaling cascade activates immune cells (158).

#### 4.1.1. Dectin-1

It is a type II trans-membrane protein receptor (C-type lectin receptors, CLRs), and its structure consists of four parts such as (1) a carbohydrate recognition domain, (2) a single trans-membrane region, (3) a short stalk region, and (4) a cytoplasmic tail consisted of immunoreceptor tyrosine-based activation motif (ITAM) (159–161). It is expressed in macrophage, dendritic, and neutrophil cells, which are responsible for an innate immune response (149, 162). Dectin-1 recognizes explicitly and binds *β-*(1,3) and *β-*(1,6) glucans from bacteria, seaweeds, fungi, and plants (142, 160, 163). The binding of Dectin-1 with *β-*glucans can start and control the innate immune response (142, 162), such as phagocytosis, inflammatory cytokines production, ROS production, and pro-inflammatory factors production, leading to the elimination of infectious agents (158, 164, 165). Dectin-1 contains six cysteine residues among 244 amino acids, particularly Trp221 and His223 are situated close to the fourth cysteine residue, which is especially important for *β-*glucan binding (166–168). On the cytoplasmic tail, an ITAM-like motif (YxxI/Lx7YxxL) communicates through the spleen tyrosine kinase (Syk) in cooperation with TLR 2 and 6 (161).

Upon *β-*glucan binding, Src family kinases phosphorylate the tyrosine in the ITAM sequence *via* interacting with Syk’s two SH2 domains (Src homology 2) (169). For the enzyme activation, the YxxL sequences must be spaced apart to engage both of the SH2 domains of Syk family kinase (Figure 5). It has been known that Dectin-1 multimerizes upon ligand binding and then provides a binding site for the Syk kinase (170). The recruited Syk activates the nuclear factor kappa-light-chain-enhancer of activated B cells (NF-κB) and CARD9-Bcl10-MALT1 pathways to induce dendritic cell maturation, co-stimulatory molecules, and inflammatory cytokines (171). Additionally, it also promotes Th1 and Th17 responses to arrange immunity to pathogens (172). Ligand-binding Dectin-1 activates phospholipase Cγ *via* phosphorylation and then activated phospholipase Cγ generates inositol trisphosphate and diacylglycerol for triggering an intracellular Ca^2+^ flux in dendritic cells (173). Elevated concentration of Ca^2+^ is crucial for secreting IL-2, IL-6, IL-10, IL-12, IL-23, and TNF α. Dectin-1 also modulates the expression of cytokines *via* activating the nuclear factor of activated T cells (NFAT) that regulates IL-2, IL-10, and IL-12 p70 production (174).

A 2.8 Å high-resolution crystal structure of murine Dectin-1 was obtained with a laminaritriose and revealed higher order complex formation between Dectin-1 and *β-*glucans (142). It comprises two antiparallel β-sheets and two α-helices with domain integrity maintained by three disulfide bridges. It has been postulated that hydrophobic contacts might play a key role in *β-*glucan binding (142, 175). Alanine mutations confirmed that Trp221 and His223 at the surface groove are critical in the formation of the *β-*glucan binding site on Dectin-1, and this site finds to be conserved among Dectin-1 of murine, chimpanzee, rhesus monkey, cow, and humans (168). It was further proposed that a minimum length of the ligand should be 10 to 11 of *β-*linked glucose residues (163), and Dectin-1–*β-*glucan complex might get more robust in the presence of divalent ions (142). Takano et al. (176) observed that low-valency β-glucan (such as fucan, a seaweed) only activates human Dectin-1 but not murine Dectin-1, and this specificity is determined by intracellular domain rather than a ligand-binding domain. Therefore, a complex structure of β-glucan can activate both types of Dectin-1.

A previous study by Brown, O’Callaghan (142) theoretically suggested that CTLD of Dectin-1 undergo oligomerization and form a quaternary structure when ligand binding to CTLD. It was proposed based on the Syk kinase’s binding to the cytoplasmic parts of two nearby Dectin-1 monomers as part of a signaling pathway. Dulal et al. (177) further reinforced this evidence by demonstrating laminarin binding. The study observed that it forms a tetramer of CTLD when four laminarin molecules bound to four CTLD cooperatively. The formation of oligomerization seems to be physiologically relevant in triggering intracellular signaling. This formation is quite appropriate for eliminating fungal pathogens through phagocytosis and triggering a pro-inflammatory immune response. This reckoning can be used for the rational design of *β-*glucans-based immunomodulatory therapy.

#### 4.1.2. TLRs

TLRs are the vital mediators of inflammatory pathways in the gut that play a major role in orchestrating the immune responses to a wide range of PAMPs and the link between innate immunity and adaptive immunity. TLRs are the type I transmembrane receptors that belong to glycoproteins. TLRs have three domains as follows: (A) an intracellular Toll-interleukin 1 receptor (TIR) domain, which is essential for downstream signal transduction, (B) a single transmembrane domain, and (3) an extracellular domain (consisting of leucine-rich repeats) that recognizes specific PAMPs (178). They are present in dendritic cells, endothelial cells, macrophages, B cells, and T cells. Microbes such as bacteria, fungi, viruses, and protozoa can get recognized by TLRs (179). The ligand-receptor binding activates several signaling pathways, including TRIF-mediated and MyD88-mediated signaling that are associated with the recruitment of neutrophils through fast mobilization (180). TRIF-mediated and MyD88 signaling also cause NF-κB activation and MAPK signaling (131, 181). NF-κB is a predominant transcription factor, which is intricate in the TLR-mediated production of cytokines (Figure 4).

*β-*Glucans modulate the signaling of TLR2 and TLR4 (182). It was found that *β-*glucans suppressed TNF-α and IL-6 production by microglia *via* binding to Dectin-1 (183). Zymosan binding with Dectin-1 enhances TLR2/4/6-mediated production of IL-10, TNF-α, and ROS through NF-κB signaling from macrophage and dendritic cells (182). Dectin-1 activation by particulate *β-*glucans was shown to promote the production of pro-inflammatory cytokines IL-6, IL-8, and TNF-α in THP-1 macrophages *via* co-binding with TLR2 and TLR4 (184), as also shown in Figure 4. The study also suggested that particulate *β-*glucan exhibited a stronger immune response than soluble. In addition to zymosan, barley-*β-*glucan is also interacted with TLR2 and Dectin-1 and induces inflammatory responses in *Leishmania donovani-*infected macrophages (158). Thus, these studies demonstrated that *β-*glucans are an immune regulatory ligand for TLR2 and TLR4 and can be manipulated in the clearance of pathogens.

#### 4.1.3. Scavenger receptor

They are a family of proteins with a variety of structural variations and various biological activities. SRs are expressed on endothelial, epithelial, and myeloid cells (185). SR is classified into classes A, B, D, E, F, G, H, and I based on their structures (186). Numerous ligands including HDL (187), LDL (188), selected polyanionic compounds of microorganisms (189), and *β-*glucan recognized by SR (190). These receptors were initially described as mediating cholesterol uptake in cultured macrophages but can be reprogrammed to kill tumor cells (191, 192). Yeast *β-*glucan can be recognized by SR type A and increased their uptakes in macrophages (J774 cells) (193). Kim (194) studied SR type B1 for phagocytosis of *Coriolus versicolor* and observed that SR-B1 is not mandatory for uptaking this fungus. The binding of *β-*glucan by SR affects the polarization of adaptive immune responses; however, the proper mechanism of recognizing *β-*glucan by SR has to be known yet.

#### 4.1.4. Lactosylceramide

Lactosylceramide (LacCer) (CDw17 and Gal4Glc1Cer) is highly expressed on the plasma membranes of human neutrophils and indispensable for many cellular processes, including innate immune functions, as they act as PRR (195). It comprises a hydrophobic ceramide and a hydrophilic sugar moiety. LacCer recognizes numerous microorganisms and pathogens, including fungi such as *Saccharomyces cerevisiae, Candida albicans,* and *Cryptococcus neoformans* (196, 197). It is also identified as a *β-*glucan receptor (198). *β-*glucans isolated from *Candida albicans* encourage chemotaxis of neutrophils through LacCer-enriched microdomains (197). Under *in vitro* circumstances, the interaction of LacCer with *β-*glucan caused various cellular responses (199). *Pneumocystis carinii* isolated *β-*glucan can induce the production of macrophage inflammatory protein-2 and TNF-α *via* NF-κB and PKC signaling pathways in alveolar neutrophils (200). It can also enhance anti-microbial properties by increasing myeloid progenitor proliferation and the neutrophil oxidative burst response (131). CDw17 can bind with *β-*glucan of *Candida* and promotes their non-opsonized phagocytosis through neutrophils (201). Overall, LacCer plays a vital role in the protection against fungal pathogens.

#### 4.1.5. CR3

Activated CR3 (also called CD11b/CD18) mediates another mechanism of *β-*glucan. They are exclusively expressed in natural killer (NK) cells, macrophages, and neutrophils (202). CR3 is the major receptor on human neutrophils for *β-*glucan (202). The two chains that make up the heterodimeric transmembrane integrin CR3 are CD11b (α m) and CD18 (β2). CD11b contains two binding sites in which the C terminus of CD11b contains a binding site for *β-*glucan, while iC3b (cleaved component 3 fragment of serum complement system) attaches within the N-terminus of it (203). CR3 is peculiar among other integrins in consisting of a lectin-like domain that binds *β-*glucan of the fungal pathogen and assists as the central receptor for reckoning fungal pathogens by human granulocytes. When *β-*glucan binds to the C-terminal lectin-binding domain, it increases adherence of microbial cells and activates iC3b pathways that cause tumor cytotoxicity (204). Additionally, numerous cellular responses including adhesion, cytotoxicity, phagocytosis, and migration (205) mediate upon ligand and CR3 binding (206). The CR3-containing neutrophil and circulating cells have stimulated by *β-*glucans that cause cell lysis on iC3b-coated tumor cells (207). Thus, CR3 may provide an alternative way for developing therapeutic *β-*glucans for the clearance of tumor cells and fungal pathogens. Interestingly, CR3 is also recognized in low MW (1,3)-*β-*glucans, generated from high MW (1,3)-*β-*glucans through the actions of macrophages and other cells. CR3 was initially anticipated to be the main (1,3)-*β-*glucan receptor on leukocytes but the ability of CR3-deficient leukocytes to still reckon and respond to (1,3)-*β-*glucans and the discovery of Dectin-1 suggests that CR3 may only display a minor role for macrophage and dendritic cells (reference herein).

Based on immunological studies, *β-*glucans considered active compounds to induce immune effects and initiate anti-microbial immune responses and anti-tumor activities. *β-*glucans emerged as an effective immunomodulatory as it acts on various immunological receptors, namely, Dectin-1, CR3, LacCer, SR, and TLR-2/6. It triggers immune cells such as macrophages, neutrophils, dendritic cells, monocytes, and natural killer cells. These results induce several immune reactions against the pathogen, such as phagocytosis, inflammatory cytokines production, ROS production, and pro-inflammatory factors production. Overall, these lead to the elimination of infectious agents. Thus, *β-*glucans are essential in controlling the host’s immunity, resulting in a healthy individual.

## 5. Biological application of *β-*glucans

### 5.1. *β-*Glucan impacts epithelial integrity *via* gut microbiota

The gut microbiota impacts epithelial homeostasis and is known to encourage epithelial integrity and proliferation. The integrated relationship of gut microbial communities provides the host with structural, metabolic, and protective functions, necessary for sustenance. *In vitro* study found that the fermentation of barley and oat *β-*glucan by human fecal samples show variations in SCFAs production and the bacterial populations of *Clostridium histolyticum* and the ratio of *Bacteroides–Prevotella* species (88, 208, 209). Absorption of these SCFAs by the gut epithelial cells helps in regulating cell differentiation, proliferation, apoptosis, and gene expression (210). Butyrate increases the protein expression of tight junctions such as ZO-1 and claudin-1, resulting in enhanced intestinal barrier function (211).

### 5.2. *β-* Glucan lowers the level of cholesterol

The effects of *β-*glucans in reducing cholesterol are widely accepted. The soluble *β-*glucans help in various activities such as lowering the total level of low-density lipoprotein (LDL), preventing the transit of triglycerides and cholesterol across the gut (Table 3), and prolonging gastric emptying by forming viscous solutions (227). Diet enhanced with *β-*glucan-rich grain affirmed the hypocholesterolemic impacts of glucans in the broiler chicks (228). A high-fat meal was used to increase the production of *β-*glucans from the *Aureobasidium pullulans* in the hamster experimental animal model of hyperlipidemia (212). A subsequent study showed that glucan lowered triglyceride levels, total cholesterol by 32% and malondialdehyde levels by 45% (229). LDL and total cholesterol levels considerably decreased when Granoro’s Cuore Mio pasta was supplemented with barley-β-glucans (3 g/100 g) (230). Supplementing with oat *β-*glucans decreased the amounts of LDL and very LDL by 25–31% and 0.2–2.3%, respectively. It also reduced total cholesterol and triglyceride levels and increased the high-density lipoprotein, HDL (231). Oat *β-*glucans lower cholesterol through gut microbiota by producing SCFA, particularly propionate. As the ratio of propionate to acetic acid (the primary substrate for cholesterol production) rises, the rate of cholesterol biosynthesis declines (232). Concerning this, an intriguing study has highlighted that in Caco-2/TC-7 enterocytes, propionic and butyric acids decreased the mRNA levels of 3-hydroxy-3-methylglutaryl-CoA reductase (HMG-Co-A), the rate-limiting enzyme of cholesterol production (233).

**Table 3:** Studies for evaluating impact of *β-*glucan on colorectal cancer, diabetes mellitus, cholesterol, epithelial integrity, and inflammation.

*β-*Glucan	Model system	Impact	References
*In vivo studies*			
Polycan (*Aureobasidium pullulans*)	Male hamsters (age, 7 weeks)	Decreases HFD-induced hyperglycemia and associated atherosclerosis, with relatively good protective effects on liver damage.	Lim et al. (212)
Oat-*β-*glucan	Clinical study in patients	Increases *Akkermansia muciniphila* and leads to reduction of total cholesterol	Li et al. (213)
Barley-*β-*glucan	Adult male Sprague–Dawley rats	Reduction in colon inflammation	Kopiasz et al. (214)
Yeast *β-*glucans	Male C57BL/6 J mice	Improves insulin sensitivity and hepatic lipid metabolism	Mitchelson et al. (215)
Glucooligosaccharides (GOS)-supplemented HFD	C57Bl/6 male mice	Change in gut microbiota population that shows an essential role of GOS in controlling diabetic metabolic phenotype.	Serino et al. (216)
Barley-*β-*glucan	50 healthy subjects without a prior diagnosis of diabetes mellitus were included. Out of them, 44 were completers who were administered beverages containing placebo (control), a lower dose (3 g/d), or a higher dose (6 g/d) of reduced viscosity barley-β-glucan extract.	Improve insulin sensitivity among hyperglycemic individuals with no prior diagnosis of diabetes mellitus.	Bays et al. (217)
Oat-*β-*glucan	A total of 100 free-living hypercholesterolemia subjects were locally recruited and 89 completed the study.	Increases the population of *Bacteroides–Prevotella* species and propionate and butyrate ratio during *in vitro* studies. It improves insulin levels and maintains glucose homeostasis.	Biorklund et al. (218)
Oat *β-*glucans	A total of 16 male, well-controlled type 2 diabetes patients	A greater increase in HDL cholesterol and larger decreases in the hemoglobin A1c, weight, and body mass index were found.	Reyna et al. (219)
6% oat-*β-*glucan concentrate	Pig	Significant decreases in glucose levels and increases in the levels of SCFAs and insulin.	Braaten et al. (220)
*β-*glucan	A total of 19 adult females and males.	It reduces DNA damage substantially in colorectal cancer patients and shows anti-mutagenic effects.	Benlier et al. (221)
*β-*glucan	Mice	It significantly decreased the TNF-α level and down-regulated three genes (*hmgcs2, fabp2,* and *gpt*) that are associated with inflammation and cancer. It increases the relative abundance of *Parabacteroides.*	Qi et al. (222)
Lentinan	Clinical study in patients	Increases host defense mechanisms against murine and human tumors. Induces the production of IL-12 and the binding ability of peripheral blood monocytes. Shows a positive effect on long-term survival and the improving quality of life status.	Hazama et al. (223)
Aminated *β-*1,3-D-glucan and interferon-gamma	Syngeneic mice	Inhibited the growth of liver metastases significantly.	Sveinbjornsson et al. (224)
*β-*glucans (paramylon and its isomer amorphous paramylon)	Mice	Shows preventive effects against colon cancer.	Watanabe et al. (225)
*In vitro studies*			
Oat-*β-*glucan	Caco-2 cell line and HT29-MTX-E12 cell line	Increases 28% butyrate production that promotes the tightness of the gut barrier.	Pham et al. (36)
Barley-*β-*glucan	Caco-2 cells and dendritic cells	Reduction of proinflammatory markers in the colon	Bermudez-Brito et al. (226)

### 5.3. *β-*Glucans are effective cardio protectors through gut microbiota

Cardiovascular disease (CVD) is proven to increase drastically globally and is one of the leading causes of death. The pathogenesis of CVD is heavily influenced by microbial dysbiosis (234). Microbial communities control CVD and atherosclerosis by regulating the production of trimethylamine *N*-oxide (TMAO) (43). Trimethylamine is a precursor of TMAO, which is formed in the gut *via* peculiar bacterial choline trimethylamine (TMA) lyases. Specifically, TMA moieties (such as choline, phosphatidylcholine, and L-carnitine) containing fatty acids are converted to TMA by bacterial TMA lyase *via* various metabolic pathways (235). Formed TMA is transported to the liver, where it is converted into TMAO by hepatic flavin monooxygenase 3, FMO3 (236). A previous study showed that oat-*β-*glucan promotes the expansion of *the Verrucomicrobia* population (such as *Akkermansia muciniphila*), which has a prebiotic impact on alterations in circulatory lipids and decreases the number of plaques in the aortic walls as compared with simvastatin (is an oral antilipemic agent) (213). In addition, oral administration of live *A. muciniphila* minimizes the expansion of atherosclerotic lesion formation and systemic inflammation in the aortic as well as enhanced intestinal integrity in atherosclerotic Apoe^−/−^ mice (213). Plovier, Everard (237) used pasteurized *A. muciniphila* in mice model experiments and observed that their administration could suppress HFD-induced expression of FMO3 compared with control diet-fed mice. This evidence specifies that *A. muciniphila* protects against CVD development in live or pasteurized conditions. The *Firmicutes* to *Bactereoidetes* ratio is significantly higher in persons at risk for cardiovascular disease, in which *Prevotella* and *Klebsiella* species are abundant in assessing the fecal microbiota of atherosclerotic CVD patients (238). Overall, it has been highlighted that maintaining gut microbiota, especially *A. muciniphila* population, is essential for mitigating CVD by taking an adequate amount of *β-*glucan as a dietary supplement.

### 5.4. *β-*Glucan can regulate type 2 diabetes by promoting gut microbes

Type 2 diabetes (T2D) is a metabolic disorder that is categorized by hyperglycemia resulting from failings in insulin secretion from *β* cells of the pancreas and insufficient insulin action. This is typically characterized by symptoms such as polyuria, polyphagia, polydipsia, and weight loss (239). T2D links to a modified gut microbial population that exhibits less diversity and resilience (240).

*β* cells are responsible for insulin production, and produced insulin is stored in secretory granules. High glucose concentrations in the blood mainly trigger insulin release; however, it can also induce by the availability of fatty acids and amino acids in the blood (241). A solute carrier protein called glucose transporter 2 (GLUT2) primarily serves as a glucose sensor for *β* cells in rodents (242), while GLUT1 is suggested to take a significant role in glucose uptaking by many cells in humans including *β* cells (243). When circulating glucose level increases, *β* cells mostly absorb glucose *via* the GLUT2 (244, 245). The glucose catabolism is activated when the glucose enters into *β* cells. Cytoplasmic glucose immediately converts into phosphorylated glucose and enters into a glycolysis cycle to produce pyruvate. Pyruvate transports to mitochondria, which processes in the Krebs cycle to generate ATP. It increases the intracellular ATP/ADP ratio, stimulating the plasma membrane’s ATP-dependent potassium channels to close. It causes the membrane to depolarize and the voltage-dependent Ca^2+^ channels to open, letting Ca^2+^ into the cell. The increased intracellular Ca^2+^ causes the secretory insulin-containing granules to prime and fuse to the plasma membrane, leading to insulin exocytosis (246). Additionally, ryanodine receptors (RYR), primarily associated with the endoplasmic reticulum and on the secretory vesicles, can amplify Ca^2+^ signals and are involved in increasing the secretion of insulin when the channel is sensitized by the influx of messenger molecules (247, 248). Such a process is called Ca^2+^-induced Ca^2+^ release (CICR). Many glycolytic intermediates, such as ATP, cAMP, cyclic ADP ribose, nitric oxide (NO), long-chain acyl CoA, and high luminal Ca^2+^ concentration, have been shown to sensitize RY receptors (248). Perhaps, the most significant messenger promoting insulin released is cAMP, thereby increasing intracellular Ca^2+^ concentration (249, 250).

Chronic hyperglycemia and hyperlipidemia are vital causative factors for T2D, disrupting endoplasmic reticulum homeostasis to induce unfolded protein response (UPR) activation. If homeostasis cannot revert to customary conditions, the ER recruits death signaling pathways, which leads to *β-*cell death (251). High levels of saturated free fatty acids can cause ER stress that activates the UPR pathway by various mechanisms, such as inhibition of the enzyme that mobilizes ER Ca^2+^ (i.e., ER Ca^2+^ ATPase), activation of IP3 receptors, and/or directly impairing ER homeostasis (241). During high blood glucose levels, proinsulin biosynthesis and islet amyloid polypeptides (IAAP) are significantly increased in *β* cells. These abrupt changes in glucose levels lead to the accumulation of misfolded insulin and IAAP in *β* cells. It ultimately increases the production of oxidative protein folding-mediated reactive oxygen species (ROS) (251). Therefore, physiological ER Ca^2+^ mobilization gets altered by these effects, which favors the degradation of proinsulin mRNA and pro-apoptotic signals. ROS promotes releasing of interleukin (IL)-1, which attracts macrophages and intensifies local islet inflammation (252).

Reduction in SCFAs synthesis due to intestinal dysbiosis encourages pancreatic *β-*cell proliferation, insulin production, and glucose tolerance, showing that these impacts are dependent on short-chain fatty acid receptors FFA2 and FFA3 in mouse model system (253). Synthesis of additional metabolites, including TMA and branched amino acids, can also cause dysbiosis, disrupt glucose homeostasis, and trigger the development of T2D (241). A study including 277 non-diabetic Danish people discovered that the human gut microbiome populations have an effect on serum metabolome and are linked to insulin resistance (254). Butyrate-producing bacteria having anti-inflammatory properties such as *Clostridium*, *Roseburia*, and *Faecalibacterium* species, have reduced significantly in T2D patients, while the population of gram-negative bacteria, such as *Escherichia,* increases high levels of lipopolysaccharides, LPS. LPSs are responsible for low-grade inflammation, causing glucose metabolism abnormalities in T2D patients (255). *Prevotella copri* and *Bacteroides vulgatus* were found to be the primary species driving the relationship between the branched-chain amino acids (BCAAs) biosynthesis and insulin resistance in a Danish cohort of non-diabetic males. The study further stated that *P. copri* can cause insulin resistance, exacerbate glucose intolerance, and increase mouse circulating BCAA levels (254, 256). It suggests that intestinal microbiota could be an essential resource for increased levels of BCAAs and display a key role in insulin resistance.

Through SCFA receptor GPR43, the gut bacteria inhibit insulin-mediated fat storage. In particular, SCFA-mediated activation of GPR43 in adipocytes reduces insulin signaling, resulting in the prevention of fat accumulation and an increase in the metabolism of lipids and glucose (257). Pigs given 6% oat *β-*glucan significantly reduced blood glucose levels and increased insulin and SCFA levels (220). Products high in β-glucan can lower glucose levels and insulin responses more than those low in dietary fiber (217). The C57BI/6 mouse was fed with *β-*glucan and observed that they were evolved to have a diabetic metabolic phenotype despite possessing the same genetic determinants, suggesting that the alteration in the gut microbiota population may be a significant factor in the development of diabetic metabolic phenotype (258). *β-*glucans can play an essential role in increasing the viscosity of a meal during digestion in the intestine, slowing down gastric emptying, limiting the absorption of macronutrients, and entrapping cholesterol and bile acids (259). Thus, *β-*glucans lower cholesterol and serum sugar levels in T2D (Table 3).

### 5.5. *β-*Glucans can prevent colon cancer by modulating gut microbiota

Dysbiosis in the gut microbiota causes human colorectal cancer (CRC). CRC is the third most common type of cancer with about 2 million new cases every year, and the gut microbiome can modulate a crucial role in their progression or prevention (260). The presence of healthy or altered gut microbiomes determines the formation and progression of CRC (261). The altered gut microbiome during dysbiosis negatively impacts CRC treatments with chemotherapy and immunotherapy (262, 263). A few bacteria, namely, *Bacteroides fragilis, Fusobacterium nucleatum*, *Parvimonas micra*, *Porphyromonas asaccharolytica,* and *Prevotella intermedia*, are suggested to associate with CRC conditions (264). These bacteria can cause initial inflammation and modulate different signaling pathways for the progression of CRC (265–267). The biological action of gut microbiota interrupts the control of the cell cycle by generating genotoxins which may lead to oxidative stress and a chronic inflammatory state (268). Bacterial metabolites, such as SCFAs, can also suppress the development of CRC. Among other SCFAs, butyrate is considered an essential metabolite and plays a vital role in inhibiting colon cancer because of its capacity to renew the intestinal epithelial cells (267, 269). It improves the tight junction of the epithelial cells, thereby minimizing the translocation of bacteria and metabolites in lamina propria that trigger inflammation (270). In response to dietary intake of fiber-rich foods, species from the *Lachnospiraceae, Bifidobacteriaceae,* and *Ruminococcaceae* families produce butyrate, lowering the risk of CRC. Butyrate can reduce tumors through a variety of mechanisms, including apoptosis induction, epigenetic alteration in gene expression, reduction of cell proliferation, and manipulation of cytokine levels and inflammatory responses during *in vitro* studies (271, 272). In particular, Donohoe, Collins (273) decisively demonstrated that due to undergoing the Warburg effect, colon cancerous cells primarily rely on uptaking glucose instead of butyrate as a primary carbon source for the production of lactate. Because of that effect, butyrate continuously collects in the cells and at certain physiological concentrations, it acts as an inhibitor of histone deacetylases, leading to the death of the cancerous cells. In addition to butyrate, bacteriocins produced by gut bacteria can prevent CRC through their cytotoxic activities as it was demonstrated by clinical studies (274). Phenylpropanoid-derived metabolites are also associated with the prevention of CRC (275).

Dietary non-digestible carbohydrates enhance the protection against CRC (276). *β-*glucans, such as lentinan, schizophyllan, scleroglucan, and grifolan extracted from mushrooms, have been studied for controlling CRC *via* modulation of gut microbiota and regulation of immune genes (223, 277) (Table 3). *β-*glucans reduce the risk of CRC bt activating leukocytes, synthesizing anti-inflammatory cytokines, and activating immune cells (Figure 3). *β-*glucans were found to be an immunomodulatory agent and can be beneficial for breast cancer patients as a supplemental or adjuvant therapy (278, 279). *β-*glucans had less impact on white blood cells, significantly reducing the level of IL-4 in breast cancer patients, while IFN-γ and *β-*glucans together have completely stopped liver metastasis from growing cancerous cells (224). The frequently used chemotherapeutic medicines to prevent liver metastases are 5-fluorouracil and mitomycin. These performed better when used in association with lentinan (a *β-*glucan) as compared with what they did when used separately. Thus, a better understanding of the roles of *β-*glucans in preventing cancer at the mechanism level would be helpful in developing nutraceutical therapy.

## 6. Conclusion

*β-*glucan is an essential food ingredient in controlling metabolic dysregulations linked to metabolic syndrome. Nevertheless, the impact of *β-*glucan is shaped by their dose, style, MW, and glucoside linkage. Given the intimate symbiotic link between the host and the gut microbiota, it is not surprising to see a divergence from the typical microbiota composition (usually referred to as dysbiosis) in a variety of illness states, ranging from chronic GI diseases to neurodevelopmental disorders. Additionally, *β-*glucans have a very minimal probability of having any unfavorable side effects and are reasonably inexpensive. Human gut bacteria display diverse molecular mechanisms for utilizing those *β*–glucans and support other bacteria that cannot utilize complex structural *β*-glucans. The impacts of *β-*glucan on different diseases, such as cancer, diabetes, cardiovascular, and low immunity, have been examined by several researchers. Notwithstanding, how *β-*glucan exerts these many biological actions at the defined and molecular levels is still unclear. Perhaps, immunostimulation may be the initial mechanism governing the *β-*glucan activity. Specifically, binding of *β*-glucan to certain receptors in cells such as macrophage and dendritic cells can trigger the production of different cytokines, which indirectly activates other immune cells, including T and B cells in *in vivo* setting. The primary method for inhibiting the development of cancer cells and infectious microorganisms in the host may involve systemic immunostimulation. Many *β-*glucan receptors in macrophages and dendritic cells including Dectin-1 and TLRs are essential for recognizing *β-*glucans, but the precise signaling pathways that lie downstream from each receptor are unknown. Future research should seek to gather this knowledge to help us to use *β*-glucans to treat future patients rationally and efficiently.

## Author contributions

AB and RS designed this research, collected different articles, wrote, edited, and reviewed the manuscript. Both authors contributed to the article and approved the submitted version.

## Conflict of interest

The authors declare that the research was conducted in the absence of any commercial or financial relationships that could be construed as a potential conflict of interest.

## Publisher’s note

All claims expressed in this article are solely those of the authors and do not necessarily represent those of their affiliated organizations, or those of the publisher, the editors and the reviewers. Any product that may be evaluated in this article, or claim that may be made by its manufacturer, is not guaranteed or endorsed by the publisher.
